# Study on Mechanical and Frost Resistance Properties of Slag and Macadam Stabilized with Cement and Fly Ash

**DOI:** 10.3390/ma14237241

**Published:** 2021-11-26

**Authors:** Hongbo Li, Pengfei Yan, Juncang Tian, Hao Sun, Jianguang Yin

**Affiliations:** 1 College of Civil and Hydraulic Engineering, Ningxia University, Yinchuan 750021, China; lhbiongo@126.com (H.L.); 13469578416@nxu.edu.cn (J.T.); sunhaoshine@csu.edu.cn (H.S.); yinjianguang777@163.com (J.Y.); 2 Ningxia Center of Research on Earthquake Protection and Disaster Mitigation in Civil Engineering, Yinchuan 750021, China; 3 Ningxia Research Center of Technology on Water-Saving Irrigation and Water Resources Regulation, Yinchuan 750021, China; 4 State Key Laboratory for Geomechanics & Deep Underground Engineering, China University of Mining and Technology, Xuzhou 221116, China; 5 School of Civil Engineering, Central South University, Changsha 410075, China

**Keywords:** slag and macadam stabilized with cement and fly ash, mechanical performance, frost resistance, ultrasonic testing

## Abstract

China is a large country in terms of coal production and consumption. The fly ash and slag produced by thermal power plants pose a great threat to the environment. To reduce the adverse effects of fly ash and slag on the environment, a mixture of slag and macadam stabilized with cement and fly ash was prepared as pavement base material. Compaction tests, unconfined compressive strength tests, splitting strength tests, frost resistance tests, and ultrasonic tests were performed on the mixture. The results show that with an increase in slag replacement rate, the unconfined compressive strength and splitting strength decreased. However, the adverse influence of the slag replacement rate on unconfined compressive strength and splitting strength of specimens gradually weakened with increasing curing time. The frost resistance of the mixture first increased and then decreased with an increase in the slag replacement rate. When cement content was 5% and the slag replacement rate was 50%, the frost resistance of the mixture was the best. Regression analysis of the ultrasonic test showed that the ultrasonic test can effectively characterize the strength of the mixture and the internal damage degree under freeze–thaw cycles. In conclusion, the slag replacement rate of the mixture is recommended to be ~50%, which has preferable mechanical and frost resistance performance.

## 1. Introduction

Coal is the primary energy source for thermal power generation in China. According to available data, ~5.2 trillion kW·h of thermal power was generated in 2019. Approximately 140 million tons of fly ash and slag are generated per trillion kW·h of power generation. Therefore, the amount of fly ash and slag produced by thermal power generation was ~730 million tons in 2019. Compared with the developed countries in the west, the high value-added utilization rate of coal fly ash and slag in China has always been lower. Generally, fly ash is used only as roadbed backfill or in admixtures of concrete and blocks. In Ningxia, Inner Mongolia, Guizhou, and Yunnan, most of the fly ash and slag produced is discharged directly as waste, which not only causes surface and groundwater pollution but also occupies considerable amounts of valuable land resources. This pollution has a severe effect on the quality of life for the surrounding residents and limits the local economic development. Thus, the potential value of fly ash and slag has been extensively explored in previous studies to realize the resource utilization of industrial solid waste [1,2,3,4,5,6,7,8]. Using fly ash and slag as pavement base materials has broad application prospects and significant advantages. For example, no special requirements are indicated regarding the type and quality of fly ash and slag for this application, and harmful compositions are not restricted. Moreover, there are no special requirements for the physical and chemical standards of fly ash and slag, which can be applied without special technical treatment; hence, most types of fly ash and slag can be used as pavement base materials. The application of fly ash and slag in road construction can consume a large amount of these industrial solid wastes and reduce environmental pollution. Therefore, abundant research has been conducted on this topic.

Xiao et al. [9] studied the influence of the ratio of lime to fly ash on the road performance of recycled base material through mechanical property tests, which revealed that the mechanical performance was best with a lime to fly ash ratio of 1:3. Zhang et al. [10] studied the durability of cement modified with a lime and fly ash–coal gangue mixture and determined that a cement content of 4% is needed to meet the requirements of strength and frost resistance outlined by related standards. Li et al. [11] studied the mechanical performance and dry shrinkage of alkali-activated fly ash cement in stabilized recycled aggregate and found that the mechanical performance of the mixture was the best when the content of fly ash was 25% and that the dry shrinkage of the mixture decreased with an increase in the fly ash content. Alireza et al. [12] studied the effects of fly ash content on the mechanical performance of stabilized macadam, brick, and recycled asphalt at room temperature and at 40 °C. The results showed that the best mechanical performance was achieved with a fly ash content of 15% and that high-temperature curing conditions improved the strength of the specimens. Thi et al. [13] studied the effects of various fly ash contents on a cement-stabilized base and found that a content of 10%–20% is needed to realize excellent mechanical properties and to meet the related standard requirements.

Compared with the characteristics of fly ash, slag has larger particles and a porous structure and generally cokes into agglomerates. This material is used in production after being crushed, sieved, and cleaned. Although slag calcined at high temperatures contains lower amounts of harmful substances, the large amount of stacked slag requires abundant land resources and affects plant growth [14]. To reduce the adverse effects of slag and the consumption of natural sand and macadam, Liu et al. [15] prepared a cement-stabilized slag–natural macadam mixture by replacing the natural macadam with various proportions of slag with grain sizes in the range of 0–9.5 mm. The mechanical and shrinkage properties of the mixture were studied. After comprehensive consideration, the mass fraction of slag replacing the macadam should be 20–30%. Xue et al. [16] replaced part of the graded macadam with municipal solid waste incineration slag. The results showed that 20% slag and 4% cement meet the requirements of the relevant specifications. Zhang et al. [17] studied the strength and damage characteristics of cement-stabilized slag and macadam through mechanical property analysis and ultrasonic tests. The results showed that the ultrasonic wave velocity is proportional to the strength of the material and that the addition of slag reduces the strength of the material. In particular, the specifications were still attained with a slag content of 35%. Liang et al. [18] studied the effects of lime dosage, lime to slag ratio, porosity, and curing time on the unconfined compressive strength of a pavement base. The strength increased rapidly during the first 7 days when all of the parameters except porosity increased.

**Figure 1:**
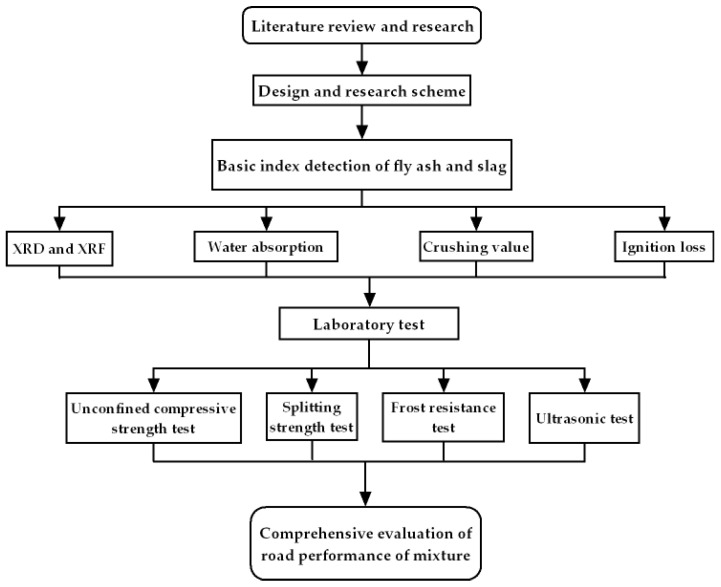
Flowchart diagram of our study.

Therefore, the numerous studies on fly ash or slag as a single admixture have achieved good results. In contrast, relatively little research has been conducted in which the 0–4.75 mm macadam is replaced by slag and is mixed with fly ash as a pavement base material. To explore the influence of fly ash and slag on the road performance of the pavement base mixture, the fly ash and slag produced by the Ningxia Xixia thermal power plant were taken as the research objects to investigate slag and macadam stabilized with cement and fly ash mixtures. Ultrasonic testing was employed to evaluate the mechanical performance, including the unconfined compressive strength, splitting strength, and strength reduction law under freeze–thaw cycles. The objectives of this study are to improve the mechanical properties and frost resistance of traditional cement-stabilized macadam base by adding fly ash and slag, reduce the consumption of macadam, and achieve the purpose of sustainable development by turning waste into treasure. The flow chart of our study is shown in Figure 1.

## 2. Materials and Methods

### 2.1. Materials

The materials used for slag and macadam stabilized with cement and fly ash mixture are as follows:

1. Cement: P·O 42.5 R Ordinary Portland Cement obtained from Ningxia Horse Racing Cement Co., Ltd., Yinchuan, China;
2. Fly ash: grade III fly ash produced by Thermal Power Plant in Xixia District, Yinchuan, China;
3. Slag: slag produced by Thermal Power Plant in Xixia District, Yinchuan, China;
4. Macadam: macadam from Helan Mountain, Yinchuan, China;
5. Water: tap water.

**Figure 2:**
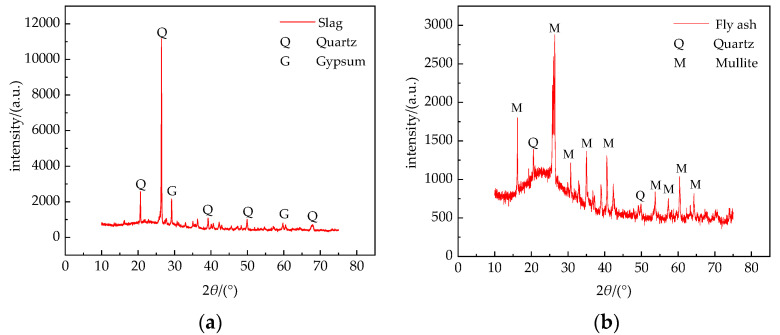
X-ray diffraction patterns of (**a**) slag and (**b**) fly ash.

The main mineral and chemical compositions of the slag and fly ash were detected by X-ray diffraction (XRD) and X-ray fluorescence (XRF). The XRD test results of the slag and fly ash are shown in Figure 2, and the XRF test results of slag, fly ash, and cement are shown in Table 1. The tests were conducted to evaluate the crushing value, apparent density, packing density, and water absorption rate of 0–4.75 mm slag and graded macadam with four grades of particle size, as shown in Table 2. The performance indicators of the cement are shown in Table 3.

Table 1 shows that the main components of the fly ash and slag are SiO_2_, Al_2_O_3_, and Fe_2_O_3_ with small amounts of alkaline oxides such as CaO and K_2_O. The mass fraction of SiO_2_ and Al_2_O_3_ in the fly ash is relatively large, accounting for ~78% of the total mass, and the loss-on-ignition is less than 10%, which meets the standard for pavement base filling materials. The mass fraction of SiO_2_ and Al_2_O_3_ in the slag is ~70%, and the alkaline oxide accounts for ~14% of the total. Therefore, the slag has high activity and is weakly alkaline. The slag is composed mainly of glass matrix and nCaO·SiO_2_. Under the action of water, these alkaline oxides react with SiO_2_, Al_2_O_3_, and Fe_2_O_3_ to show hydraulic gelling properties [19,20,21,22]. Slag and fly ash are suitable as pavement base materials when considering the chemical reaction process and the specimen strength formation mechanism. The slag has a crushing value of 38.4%, which is 19.5% greater than the average crushing value of macadam. Additionally, the water absorption of slag is much higher than that of macadam, which is determined by the porous and uncompacted structure of slag. Table 2 shows that the water absorption and crushing value of macadam are smaller than those of slag, indicating that the compactness of crushed stone is higher than that of slag and has better road performance.

### 2.2. Mix Proportion

**Figure 3:**
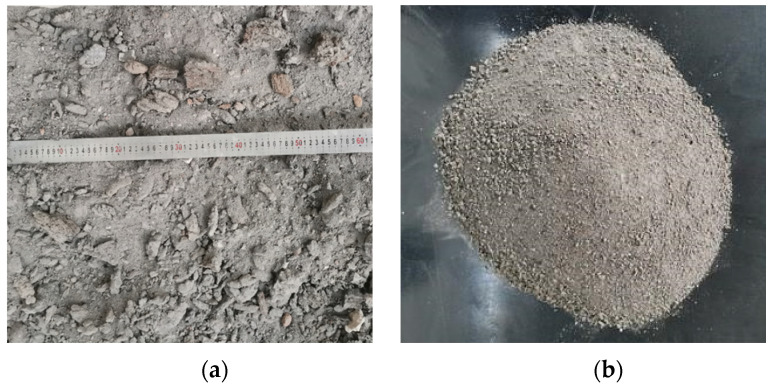
(**a**) Natural slag and (**b**) screened slag.

The natural particle-size distribution of slag is uneven and varies greatly, as shown in Figure 3a. If slag is mixed directly with other materials, it will result in a large error in test results. To reduce test errors and the consumption of macadam, 0–4.75 mm slag was used to replace the 4.75 mm macadam. The screened slag is shown in Figure 3b. According to the standard Code for Construction and Quality Acceptance of Road Works in City and Town (CJJ1-2008), the macadam gradation was divided into four grain size categories: 0–4.75, 4.75–9.5, 9.5–16, and 16–26.5 mm. After considering previous research, mixtures with five different slag replacement rates were selected in the test. After obtaining the optimal slag replacement rate, the economic cement content was obtained by taking cement as a variable. The results of the mixture design are shown in Table 4.

### 2.3. Test Methods

**Figure 4:**
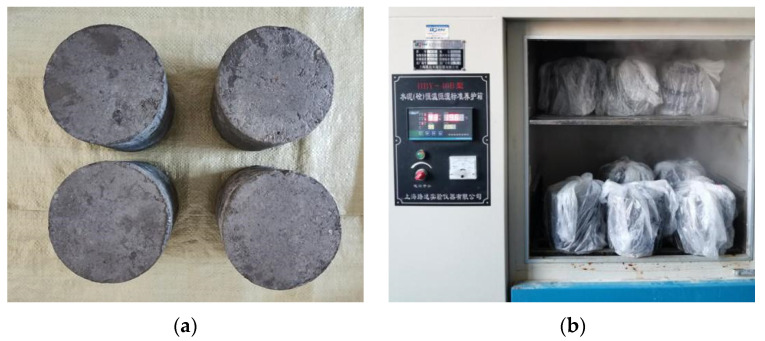
(**a**) Prepared specimens and (**b**) curing of specimens.

For the preparation and curing of specimens, a compaction test was conducted to obtain the optimum water content and maximum dry density of the mixture. Then, cylindrical specimens with a diameter to height ratio of 1:1 were prepared by the static pressing method. The diameter of the specimens was 150 mm, and the compaction coefficient was 0.98. The prepared specimens were wrapped in plastic bags and cured to the corresponding age under standard curing conditions, which were used to test the mechanical properties and frost resistance. The prepared specimens and curing environment are shown in Figure 4.

**Figure 5:**
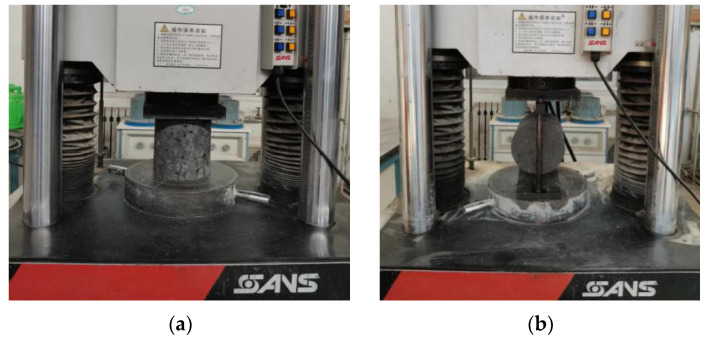
(**a**) Unconfined compressive strength test and (**b**) splitting strength test.

The unconfined compressive strength and splitting strength of the specimens were tested according to the Test Methods of Materials Stabilized with Inorganic Binders for Highway Engineering (JTG E51-2009) [23]. The test included four ages of 7 days, 28 days, 56 days, and 90 days. The tests were conducted using a Shanghai New Sansi universal testing machine. The loading rate was 1 mm/min during the test. The test setups are shown in Figure 5.

To evaluate the frost resistance, the specimens cured for 28 days were tested by freeze–thaw cycles. When cured for 27 days, the specimens were soaked in water for 1 day. Then, the immersed specimens were placed in a constant temperature testing machine at −18 °C for 16 h to ensure that a gap of ~50 mm was present around each specimen to facilitate the circulation of cold air. Then, the specimens were placed in a constant temperature flume at 20 °C for 8 h. After the ice melted, they were removed from the sink and allowed to rest for 15 min. Next, the specimens were weighed, and the average value was obtained. The unconfined compressive strength was measured for 5, 10, 15, 20, 25, and 30 freeze–thaw cycles. The frost resistance of the mixtures was comprehensively evaluated based on appearance change, mass loss, and strength loss.

**Figure 6:**
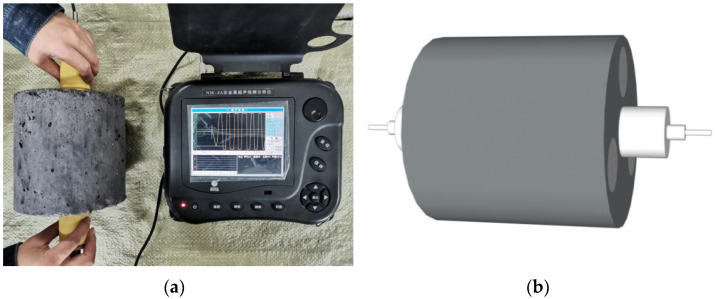
(**a**) Ultrasonic test and (**b**) simulation diagram of the ultrasonic test.

Ultrasonic testing was conducted using a nonmetallic ultrasonic analyzer (NM-4A, Beijing Concrete Co., Ltd.) with a sound time accuracy of ±0.05 μs. After setting the sound time to 0, Vaseline petroleum jelly was applied to the transmitting and receiving transducers as a coupling agent. Then, the transducer was affixed to both sides of the specimen, the sampling was engaged, and the ultrasonic test results were stored. Four points were measured on each specimen. The measuring points are indicated in Figure 6.

## 3. Results

### 3.1. Compaction Test Results

The compaction tests were conducted on samples 5-SR-0 to 5-SR-100, and the maximum dry density and optimum water content of the mixtures were obtained, as shown in Table 5.

Table 5 shows that an increase in the slag replacement rate resulted in an increase in the optimum moisture content of the mixture and a decrease in the maximum dry density. When the slag replacement rate was increased by 25%, the optimum moisture content of the mixture increased by 1.3%, and the corresponding maximum dry density decreased by 2.6%. This occurred because compared with the properties of macadam, properties of slag include a rough and uneven surface, high porosity, and low density, which facilitate the absorption of larger amounts of water.

### 3.2. Unconfined Compressive Strength

**Figure 7:**
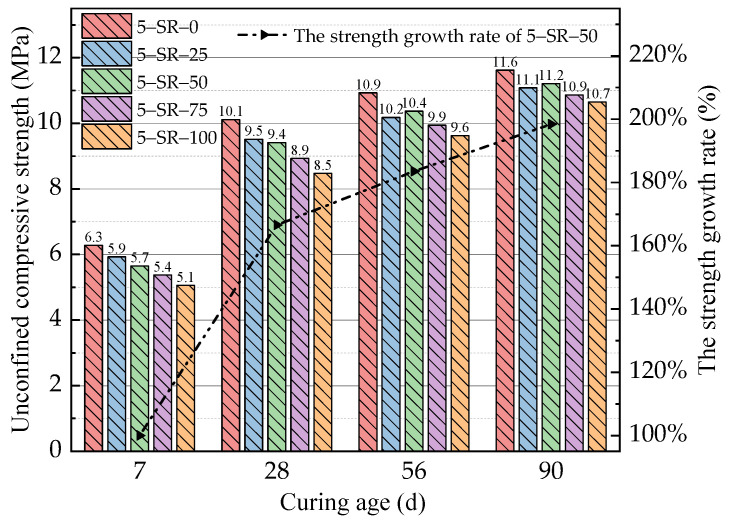
Test results of unconfined compressive strength at various slag replacement rates.

The unconfined compressive strength test results of mixtures 5-SR-0 to 5-SR-100 at different ages are shown in Figure 7.

Figure 7 shows that the unconfined compressive strength of the specimens increased with the curing age time. The unconfined compressive strength of 5-SR-0 was higher than that of the specimens mixed with slag, which shows that the addition of slag had an adverse effect on the compressive strength of the specimens. The addition of slag had an obvious effect on the early strength of the specimens. On the 7th day, the compressive strength of 5-SR-0 was 5.6%, 10.0%, 14.5%, and 19.4% higher than that of 5-SR-25, 5-SR-50, 5-SR-75, and 5-SR-100, respectively. The increase in slag replacement rate caused the unconfined compressive strength of the specimens to decrease. This occurred because the density of slag is significantly lower than that of macadam. Therefore, the density of the specimens decreased gradually with an increase in the slag replacement rate. When the slag replacement rate was 100%, the density of specimens was 10.5% lower than that of 5-SR-0. When the mass fraction of the cement was certain, the mass of cement in each specimen decreased, and the early strength of the specimen was provided mainly by cementitious substances such as hydrated calcium silicate and hydrated calcium aluminate produced by the cement hydration reaction. However, the crushing value of the screened slag was much smaller than that of the macadam, and the shape of the slag particles was mostly round with rough surfaces resembling medium sand. Compared with the 0–4.75 mm macadam, the cohesion and mechanical occlusal properties of the slag were lower. Therefore, with an increase in the slag replacement rate, the compressive strength of the specimens decreased gradually. On the 28th day, the unconfined compressive strength of the specimens was significantly higher than that on the 7th day, and the compressive strength growth rate of the different mix proportions of the specimens was more than 60%, of which the strength growth rate of 5-SR-100 was the largest (67.4%). This occurred mainly because during the extended curing age, a large number of active substances such as SiO_2_ and Al_2_O_3_ in the fly ash and slag gradually undergo secondary hydration under the excitation of the cement hydration product Ca(OH)_2_, resulting in a large number of gel substances such as C-S-H, C-A-H, and ettringite [24], as shown in the following equation:

Ca(OH)_2_ + SiO_2_ + H_2_O → CaO·SiO_2_·2H_2_O
Ca(OH)_2_ + Al_2_O_3_ + H_2_O → CaO·Al_2_O_3_·2H_2_O
3CaO·Al_2_O_3_ + (CaSO_4_·H_2_O) + H_2_O → 3CaO·Al_2_O_3_·CaSO_4_·2H_2_O (1)

These gel materials tighten the interfaces among the slag, macadam, and cement mortar, which significantly improves the internal compactness and cementation strength of the specimens. This behavior is shown directly from the improved specimen strength. On the 56th day and 90th day, the strength growth rate of the specimens decreased significantly, at an average of 16.1%; with an increase in the slag replacement rate, the increase in specimen strength was more obvious. This occurred because the amount of Ca(OH)_2_ and other active substances that participate in the reaction decreased with further increases in the degree of the secondary hydration reaction, thereby decreasing the rate of secondary hydration [25]. When the slag was used to replace the macadam, the chemical activity of the slag was higher than that of the macadam owing to the large amounts of SiO_2_, Al_2_O_3_, and alkaline oxides in the slag, which enabled the reaction of slow secondary hydration in the long curing period. With an increase in the slag replacement rate, the number of active substances in the specimens increased. Thus, more water was needed to achieve the optimum water content to provide the necessary active material and water for the secondary hydration reaction. Therefore, in the latter stage of the curing period, the adverse effect of slag on the strength of the specimens gradually decreased. On the 90th day, the compressive strength of 5-SR-0 was 4.7%, 3.5%, 6.5%, and 8.4% higher than that of 5-SR-25, 5-SR-50, 5-SR-75, and 5-SR-100, respectively. Therefore, the strength growth rate of the specimens decreased significantly during the longer curing period, and the growth was more obvious with an increase in the slag replacement rate. Moreover, unconfined compressive strength of the mixture on the 7th day meets the standard Technical Guidelines for Construction of Highway Roadbases (JTG/TF20-2015) [26]. To achieve the best replacement rate of the slag to reduce the consumption of natural macadam in practical engineering applications, a slag replacement rate of ~50–75% is suggested.

**Figure 8:**
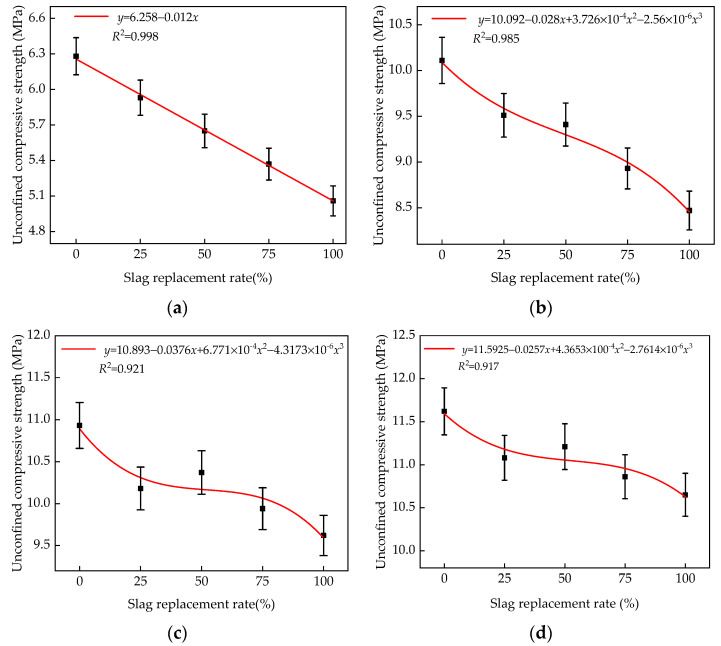
Fitting formulas of unconfined compressive strength with various slag replacement rates and ages: (**a**) 7 days; (**b**) 28 days; (**c**) 56 days; (**d**) 90 days.

MATLAB was used to fit the unconfined compressive strength formula of the different slag replacement rates at the 7th, 28th, 56th, and 90th day, as shown in Figure 8.

To study the strength development laws of the specimens with different cement contents, it is necessary to determine the optimal slag replacement rate. In this study, three principles were followed when selecting the optimal slag replacement rate. Firstly, the 7-day unconfined compressive strength loss rate of the specimens mixed with slag was controlled within 10%. Secondly, the 90-day unconfined compressive strength loss rate of the specimen was less than 5%. Finally, the slag replacement rate was increased as much as possible. As shown in Figure 7, when the slag replacements were 25% and 50%, the loss rate of the 7-day unconfined compressive strength of the specimen was less than 10%. On the 90th day, the unconfined compressive strength of 5-SR-50 was slightly better than that of other specimens, and the strength loss was less than 5%. Therefore, the optimal slag replacement rate is 50%. Apparently, the value of *x* in Table 4 is 14, and the value of *y* is 50.

**Figure 9:**
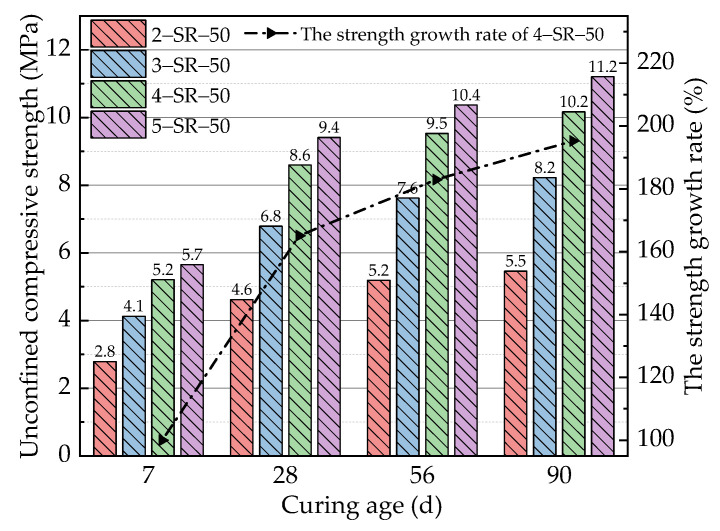
Unconfined compressive strength test results of specimens with different cement contents.

According to the obtained optimal slag replacement rate of 50%, the unconfined compressive strength values of 2-SR-50 to 5-SR-50 after 7, 28, 56, and 90 days shown in Table 4 were studied. The test results are shown in Figure 9.

With an increase in the cement content, the strength increases of the specimens varied among ages. On the 7th day, when the cement content was 2%, 3%, and 4%, the 7-day unconfined compressive strength of the specimens showed obvious increases. When the cement content increased to 1%, the unconfined compressive strength of the specimens increased to 1.3 MPa and 1.1 MPa, respectively. This occurred because with an increase in cement content, the proportion of cement clinker minerals in the specimens increases and the content of Ca(OH)_2_ increases, which rushes the hydration rate of the specimens in the early stage [27]. When the cement content increased to 5%, the 7-day unconfined compressive strength of the specimens increased only 0.5 MPa because the content of Ca(OH)_2_ in the specimens further increases with a continuous increase in the cement content. However, the content of active substances such as SiO_2_ and Al_2_O_3_, which can participate in the secondary hydration reaction, decreased at the initial stage of curing, and the reaction rate did not show an obvious increase, which directly revealed that the strength growth of the specimens slowed. On the 28th day, the unconfined compressive strength of the specimens was significantly higher than that on the 7th day, and the compressive strength growth rate with the different cement contents was more than 65%. This depended mainly on the secondary hydration reaction and microaggregate effect. On the 56th and 90th day, the strength growth rate of the specimens showed obvious decreases, and the average value was only 15.8%. With an increase in the cement content, the increase in specimen strength was more obvious. This occurred because further increases in the secondary hydration reaction caused gradual decreases in the amount of active substances such as Ca(OH)_2_, which are mainly involved in the reaction. This resulted in a slower reaction rate. In addition, the increase in cement content caused the content of Ca(OH)_2_ in the specimens to increase, and a relatively large amount of Ca(OH)_2_ still reacted with fly ash and slag in the later stage of curing. Therefore, with an increase in the cement content, the specimens can still present a relatively high strength growth rate in the later stages of curing.

**Figure 10:**
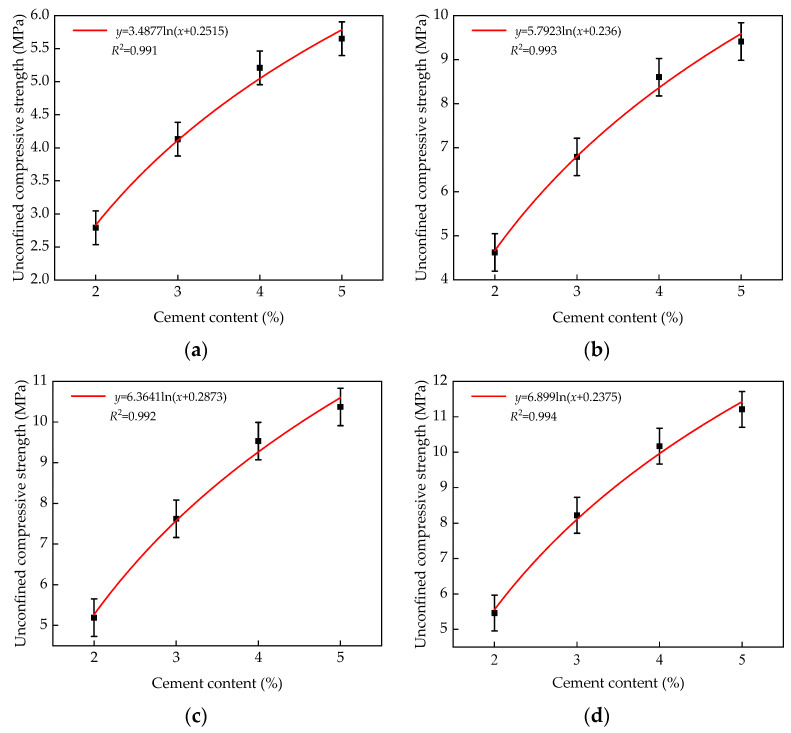
Fitting formula of unconfined compressive strength of different cement content at various ages: (**a**) 7 days; (**b**) 28 days; (**c**) 56 days; (**d**) 90 days.

MATLAB was used to fit the unconfined compressive strength formula of the different cement contents at various ages, as shown in Figure 10.

As can be seen from Figure 8 and Figure 10, the calculated values of the fitting formulas were in good agreement with the experimental values, with less than 5% error. This shows that the mixture with various slag replacement rates and cement contents at different ages can accurately reflect the relationship between slag replacement rates, cement contents, and the unconfined compressive strength. Therefore, the formulas given in Figure 8 and Figure 10 serve as the theoretical basis for practical engineering applications.

According to the unconfined compressive strength test results, the strength formation mechanism of the specimens was analyzed. The results can be summarized into three main points. First, when the slag is mixed with cement, fly ash, and macadam, the phase composition in the slag pores can be divided into three types. The first is the solid phase, including cement, fly ash, fine slag, and macadam; the second is the liquid phase, including the water molecules; and the third is the gas phase, including the unfilled area in the slag pores. With an increase in curing time, the secondary hydration reaction gradually began in the cement and fly ash in the slag pores to form the C–S–H gel material. These dense gel materials filled the slag pores that improved the high porosity and low density of the slag, which in turn improved its bearing capacity. Second, the cement, fly ash, and fine macadam filled the spaces between the slag particles and the macadam, which created a microfilling effect and improved the compactness and stability of the cement–mortar interface. Third, the fly ash and slag are high-activity substances and contain a certain amount of alkaline oxides. With an increase in the curing time, they fully reacted with the cement hydration product Ca(OH)_2_ to form dense C–S–H gel and ettringite. The formation of C–S–H gel strengthened the bond of the fly ash and slag particles with the macadam particles and improved the strength and compactness of the specimens [26]. Although the original strength of the slag was low, under the comprehensive action of the filling effect of the cement and fly ash and their inherent activity, the influence of the slag replacement rate on the compressive strength of the specimen became more complex in the later stages of curing. The compressive strength of slag is the comprehensive embodiment of all three factors.

According to the relevant standards for the 7-day unconfined compressive strength of materials stabilized with cement and fly ash, the strength representative value of 95% guarantees a pavement base rate of not less than 4.5 MPa. The strength representative value, $R_{0}^{d}$ , of the 95% guarantee rate was calculated according to the 7-day unconfined compressive strength test results. The calculated results are shown in Table 6.

As shown in the table, when the cement content was 5% and the slag replacement rate was 100% or less, the representative value of 95% guaranteed a rate strength of 4.5 MPa in the specimens, which meets the technical requirements of extremely heavy and extra-heavy traffic on expressways and first-class highways in China.

### 3.3. Splitting Strength

**Figure 11:**
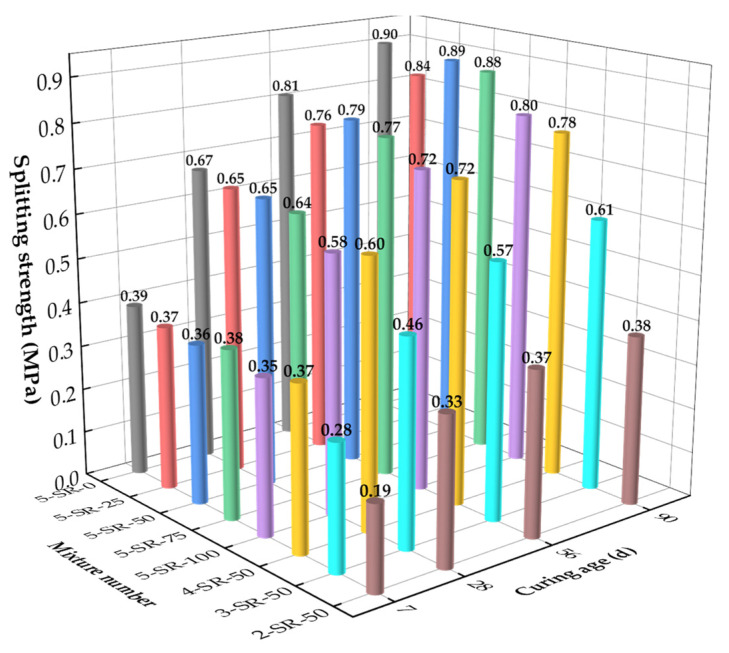
Results of splitting test with various ages.

The results of splitting test results at various ages are shown in Figure 11.

The splitting strength of the specimens increased with increases in the cement content and curing age, where the increasing law is similar to the unconfined compressive strength. The splitting strength of 5-SR-0 was higher than that of the specimens mixed with slag, which indicates that the addition of slag had an adverse effect on the splitting strength of the specimens.

On the 7th day, the splitting strength of the specimens increased with an increase in the cement content and a decrease in the slag replacement rate. The primary causes and change laws are the same as those in the unconfined compressive strength test. On the 28th day, the splitting strength of the specimens was significantly higher than that on the 7th day, and the average growth rate of the splitting strength was 70.4%. The particle-size distribution of the fly ash was very uniform, and the average particle size was much smaller than that of macadam and slag. These characteristics enable fly ash to be uniformly filled with aggregates to form a tight accumulation system, which makes the secondary hydration reaction more sufficient. However, with an extended curing age, the active substances in fly ash and slag gradually undergo secondary hydration, which consumes the Ca(OH)_2_ in the specimens, inhibits the growth of Ca(OH)_2_ grains, and reduces the thickness of the interfacial transition zone. With continued consumption of Ca(OH)_2_, the amount of the gel material increased, which further increased the splitting strength of the specimens [28].

On the 56th and 90th day, the splitting strength of the specimens with a slag replacement rate of 50% was slightly better than that with rates of 25%, 75%, and 100%. The strength growth law of 5-SR-25 to 5-SR-100 showed that an increase in slag replacement rate caused the splitting strength of the specimens to first increase and then decrease, which was determined by the failure mode of the splitting test specimens. The change rule and strength formation mechanism of splitting strength were similar to those of compressive strength, but the failure modes were different. The specimens were regarded as a composite material composed of slag, macadam, cement fly ash mortar, and transition zone. The compressive failure was caused by the expansion of cracks in the transition zone to cement fly ash mortar and the final formation of multiple main cracks. The splitting failure was caused by expansion of microcracks in the transition zone on both sides; deflection occurred when the interface transition zone was encountered, which ultimately caused the main crack to form. When the slag replacement rate increased from 25% to 50%, the interface transition zone of the micropore structure increased, the continuous deflection of microcracks consumed more fracture energy, and the splitting tensile strength improved [29]. However, with further increases in the slag replacement rate, the amount of 0–4.75 mm macadam decreased. Therefore, the pores of slag could not be filled with cement, fly ash, and fine-grained macadam, which reduced the compactness of the specimens. In addition, the density of the specimens decreased with an increase in the slag replacement rate, the proportion of cement clinker in the specimens decreased, the degree of secondary hydration reaction was reduced, and the ratio of unreacted slag and fly ash increased, resulting in deterioration of the pore structures of the specimens, thus reducing the splitting strength.

In summary, the splitting strength of the slag and macadam stabilized with cement fly ash is the comprehensive embodiment of macadam strength, the hydration reaction degree of cementitious material, and the internal cohesion of the mixture. The strength of the slag aggregate was lower than that of the macadam, which is the main explanation for the generally lower splitting strength of the slag aggregate than that of 5-SR-0. In addition, the porous and noncompact characteristics of slag promote the infiltration of cement fly ash paste into the aggregate, which enhances the compactness and stability of the specimens. In the later stages of curing, the active substances in slag and fly ash undergo secondary hydration, generating large amounts of C–A–H, C–S–H, and other cementitious substances, which improve the internal structure of the aggregate interface zone and increase the strength of the specimens. When the cement content was more than 3% and the slag replacement rate was 50%, the 90-day-splitting strength of the specimens was more than 0.4 MPa, which meets the technical index of highway pavement base material in China [15].

### 3.4. Relation between Unconfined Compressive Strength and Splitting Strength

The above test results show that the unconfined compressive strength of the specimens is positively correlated to the splitting strength, and the relationship between them has become a hot topic for researchers. However, the relationship between unconfined compressive strength and the splitting strength of pavement base materials has been rarely reported. Therefore, based on the commonly used conversion formula relating compressive strength and splitting strength for ordinary concrete (Equation (2)), the relationship between unconfined compressive strength and the splitting strength of pavement base material can be obtained.

(2) $$f_{t}=k{\left(f_{c}\right)}^{n}$$

where *f*_t_ is the splitting strength of concrete (MPa), *f*_c_ is the compressive strength of a concrete cylinder (MPa), and *k* and *n* are obtained by nonlinear regression analysis.

**Figure 12:**
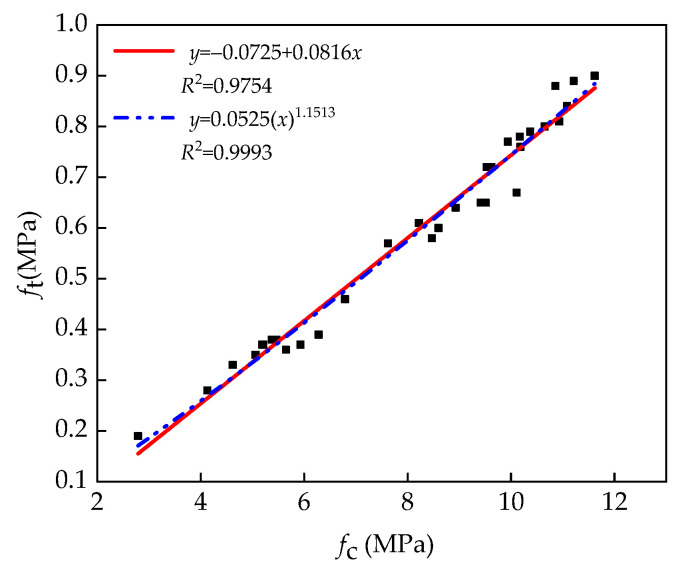
Relationship between the unconfined compressive strength and splitting strength.

An analysis of the test results revealed the relationship between unconfined compressive strength and splitting strength, as shown in Figure 12.

After fitting the test data with the power function and linear function, Equations (3) and (4) were obtained, respectively.

(3) $$f_{t}=-0.0725+0.0816f_{c}\;R^{2}=0.9754$$

(4) $$f_{t}=0.0525{\left(f_{c}\right)}^{1.1513}\;R^{2}=0.9993$$

Figure 12 shows that Equations (3) and (4) are in good agreement with the test results, and the power function is more consistent with the test results. Therefore, the power function is recommended to predict the splitting strength of the specimens at different ages to guide practical engineering applications.

### 3.5. Frost Resistance Test

#### 3.5.1. Appearance of the Specimens

**Figure 13:**
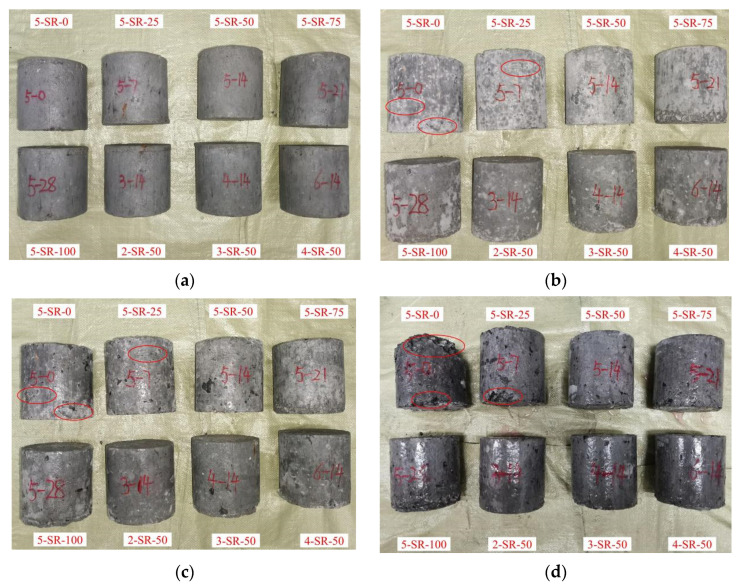
Changes in the appearance of specimens under different freeze–thaw cycles: (**a**) 0; (**b**) 5; (**c**) 15; and (**d**) 30 cycles.

The appearance of the specimens was recorded in 0, 5, 15, and 30 freeze–thaw cycles, as shown in Figure 13.

As shown in Figure 13a, the specimens had some initial defects before the freeze–thaw cycle. These defects were mainly caused by the nonuniformity of fillers in the fabrication process and the limitations of the static pressure method. After five freeze–thaw cycles, slight reticular microcracks appeared on the surfaces of 5-SR-0 and 5-SR-25 specimens, whereas there was no observable change in the appearance of other specimens. After 15 freeze–thaw cycles, the number of microcracks on the surfaces of 5-SR-0 and 5-SR-25 specimens increased and developed inward, and the fine aggregates on the surfaces of all specimens began peeling off to varying degrees, resulting in the exposure of coarse aggregates. After 30 freeze–thaw cycles, a large number of block spalling effects were observed in 5-SR-0 and 5-SR-25 specimens, as shown in Figure 13d. In addition, when the slag replacement rate was greater than or equal to 50%, little damage was evident from the appearance of the specimen, except for a small amount of fine aggregate peeling off on the surface. Massive spalling was not observed. In conclusion, when the slag replacement rate is greater than or equal to 50%, freeze–thaw cycles cause little damage to the appearance of the specimens.

#### 3.5.2. Change in the Weight of Specimens

**Figure 14:**
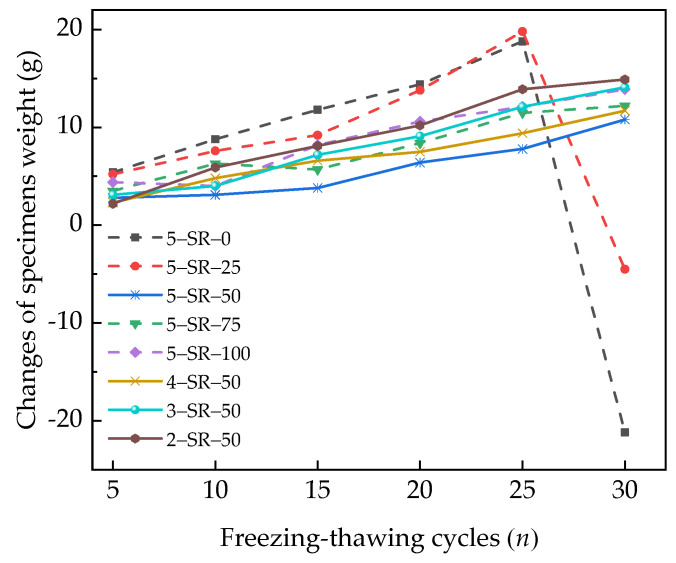
Changes in the weight of specimens under different freeze–thaw cycles.

The changes in the weights of the specimens under different freeze–thaw cycles are shown in Figure 14.

Figure 14 shows that for less than 25 freeze–thaw cycles, the weight of the specimens increases and shows an upward trend. The weight increases because after the specimens are immersed in water and saturated, the microcracks of the specimens are filled with a large amount of water. During freezing, the water inside the microcracks of the specimens changes into ice, thereby increasing the volume of the specimens and producing frost-heaving force. Hence, new microcracks form and expand in the interface transition zone of the micropore structure [30]. During melting, water fills the new microcracks, which leads to an increase in the water content of the specimens. Under the dual action of the increase in internal water content in the specimens and the exfoliation of fine aggregate on the surface, the weight of the specimens increases and shows an upward trend. As shown in Figure 13d and Figure 14, after 30 freeze–thaw cycles, massive spalling occurred on the surfaces of 5-SR-0 and 5-SR-25 specimens, and the specimen weight decreased sharply. However, the weight of specimens with a slag replacement rate greater than or equal to 50% still showed a steady growth trend. For a given slag replacement rate, the mass growth of the specimens decreases with the increase in cement content. The reason is that with an increase in the cement content, the amount of gels, such as C–S–H produced by the secondary hydration reaction, in the specimens increases. These gels envelop the surfaces of fly ash, slag, and macadam particles; hence, the interface of the transition zone tends to be dense, which in turn improves the stability and compactness of the specimens and limits the formation and expansion of new microcracks.

#### 3.5.3. Loss Rate of Unconfined Compressive Strength

**Figure 15:**
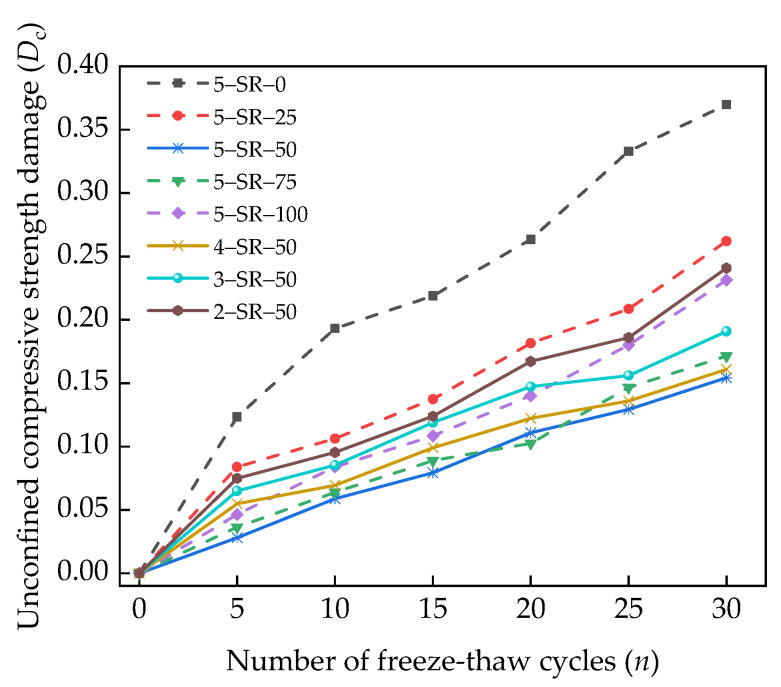
Relationship between unconfined compressive strength damage and freeze–thaw cycles.

According to reference [29], the unconfined compressive strength damage of the specimens, *D*_c_, is as shown in Equation (5). Figure 15 shows that the loss rate of unconfined compressive strength varies with the number of freeze–thaw cycles.

(5) $$D_{c}=1-\frac{P_{n}}{P_{0}},$$

where *P*_n_ is the unconfined compressive strength of the specimens under *n* freeze–thaw cycles (MPa), and *P*_0_ is the unconfined compressive strength of the specimens before freeze–thawing (MPa).

Figure 15 shows that as the number of freeze–thaw cycles increases, the unconfined compressive strength damage increases gradually. After 30 freeze–thaw cycles, the strength loss of 5-SR-0 and 2-SR-50 specimens was 37.0% and 30.1%, respectively, which is significantly greater than that of 5-SR-50 specimens (15.4%). With an increase in the slag replacement rate, the strength loss of specimens decreases significantly and then increases; this behavior is mainly determined by the porous and uncompacted structure of slag. When slag replaces a part of the 0–4.75 mm macadam, according to the analysis of the formation mechanism of the unconfined compressive strength of the specimens, the pores of slag are composed of solid, liquid, and gas phases. The gas phase in the slag is the micropore, which is equivalent to the air-entraining agent mixed in the concrete specimens. These uniformly distributed micropores effectively decrease the frost-heaving pressure formed during the freeze–thaw cycles and the osmotic pressure caused by the migration of supercooled water [31,32]. Therefore, the addition of slag significantly reduces the strength loss of the specimens. However, with the further increase in the slag replacement rate, the number of micropores in the specimens increases, leading to a decrease in the internal compactness and strength of the specimens. With an increase in the cement content, the strength loss of the specimens decreases, and the strength loss of the 5-SR-50 specimen is the least. Further, with an increase in the cement content, more gels are formed, thereby improving the pore structure of the interface transition zone and making the specimens denser. However, when the slag replacement rate is 50%, the internal particles of the specimens are in a relatively tight accumulation state, the pore composition and structure are complex, and the impermeability and frost resistance are better.

#### 3.5.4. Analysis of Freeze–Thaw Failure Mechanism

Freeze–thaw cycle failure is a physical change process of the pore water in the specimens under the alternating action of high and low temperatures. During freezing, pore water transforms into ice under the action of low temperatures, resulting in ~9% volume expansion. This leads to a certain frost-heaving force on the capillary wall. If the frost-heaving force is greater than the ultimate tensile strength of the capillary wall, the wall will break and new microcracks will form. Under the repeated action of high and low temperatures, the structure of cement paste is gradually destroyed, the width and number of cracks increase, and the pore structure of the specimen deteriorates gradually. This deterioration is characterized by the change in the weights of the specimens and the decrease in their bearing capacity.

The test results show that the addition of slag can effectively improve the frost resistance of the specimens because when slag replaces a part of the 0–4.75 mm macadam, many closed pores are formed in the slag under the microaggregate effect of cement and fly ash and the secondary hydration reaction. These closed pores resist a part of the seepage pressure, effectively reducing the invasion speed of water, damage caused by the frost-heaving force, and osmotic pressure on the specimens. Thus, the presence of the closed pores significantly improves the frost resistance of the specimens. With an increase in the cement content, the frost resistance of the specimens also gradually improves. The main reason for improvement is that with increasing cement content, the amount of dense gel materials such as C–S–H increases, the density and cohesive force of the interface transition zone of the specimens are improved, and the damage caused by the frost-heaving force and seepage pressure to the specimens is reduced.

## 4. Ultrasonic Testing

### 4.1. Relation between Ultrasonic Amplitude and Compressive Strength

**Figure 16:**
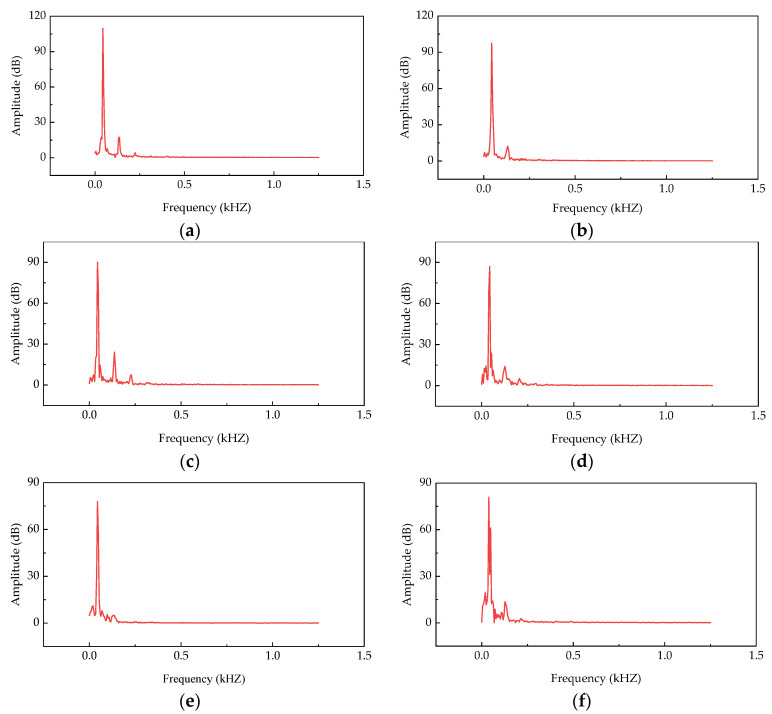

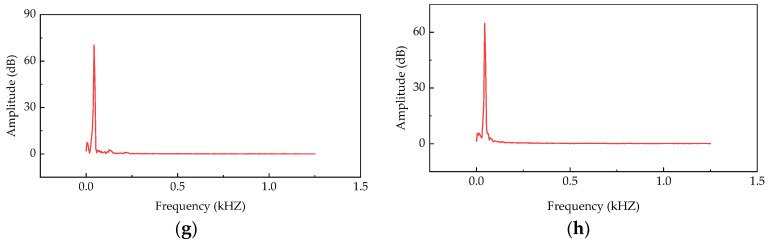
Ultrasonic testing results of specimens on the 7th day: (**a**) 5-SR-0; (**b**) 5-SR-25; (**c**) 5-SR-50; (**d**) 5-SR-75; (**e**) 5-SR-100; (**f**) 4-SR-50; (**g**) 3-SR-50; and (**h**) 2-SR-50 specimens.

The reflection and refraction of ultrasonic waves on the interface of different media can be examined to detect the compactness of specimens. In the test, ultrasonic testing was performed on the specimens on the 7th day, and the measured sound waves were converted into spectral images, as shown in Figure 16, by fast Fourier transform.

**Figure 17:**
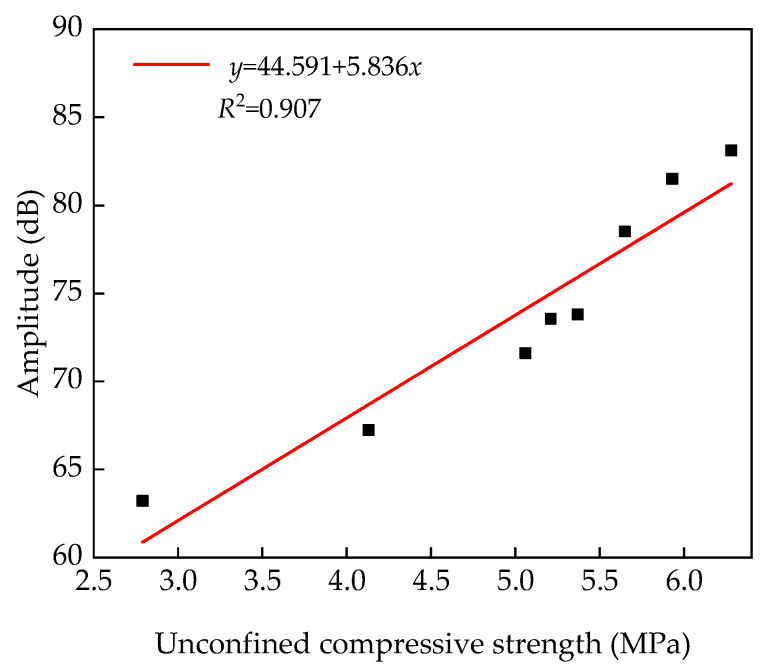
Fitting results of unconfined compressive strength and amplitude.

Figure 16 shows that the unconfined compressive strength of the specimens is positively correlated with the amplitude of the ultrasonic spectrum. The relationship between the unconfined compressive strength of the specimens and the ultrasonic amplitude (Figure 17) was analyzed. The ultrasonic amplitude was taken as the average value of the measured data.

From the fitting results shown in Figure 17, Equation (6) can be obtained.

(6) $$y=44.591+5.836x\;R^{2}=0.907$$

Figure 17 shows that the fitted results are in good agreement with the test values. Equation (6) effectively reflects the relationship between compressive strength and amplitude of the specimens, and this relation can be used to provide recommendations for practical engineering applications.

### 4.2. Relation between Ultrasonic Wave Velocity and Freeze–Thaw Cycles

Under the action of freeze–thaw cycles, the cumulative damage caused to the specimens will inevitably cause the development of cracks. Internal cracks of the specimens can absorb and dissipate the sound-wave propagation energy. Based on principle of ultrasonic wave propagation, ultrasonic rays will bypass the defect area and travel along the path corresponding to minimum travel time. Therefore, ultrasonic testing can be used to detect the internal defects of the specimens. Attenuation occurs when ultrasonic waves pass through these defects, resulting in a decrease in wave velocity [33]. Therefore, ultrasonic technology can be used to detect the damage caused to specimens under the action of freeze–thaw cycles. The measured distance, sound time, and wave velocity of the specimens are taken as the average of the measured data. The wave velocity and sound time of specimens cured for 28 days before the freeze–thaw cycles are listed in Table 7.

With an increase in the number of freeze–thaw cycles, the cracks in the specimens grow and expand rapidly, resulting in a change in the path of ultrasonic waves through the specimens. This change is directly indicated by the change in the ultrasonic sound time and wave velocity. To describe the degree of damage caused to the specimens by the freeze–thaw cycles, the relative dynamic elastic modulus, *E*_r_, is generally used [34,35,36]. Furthermore, damage factor *D* is introduced to evaluate the damage parameters. The initial damage value *D* of the default specimens is 0. The equations for *E*_r_ and *D* are as follows:

(7) $$E_{r}(n)=\frac{E_{n}}{E_{0}}=\frac{V_{n}^{2}}{V_{0}^{2}}=\frac{{\left(l/t_{n}\right)}^{2}}{{\left(l/t_{0}\right)}^{2}}=\frac{t_{0}^{2}}{t_{n}^{2}},$$

(8) $$D=1-E_{r}(n),$$

where *E*_r_ is the relative dynamic modulus of elasticity (MPa), *E*_n_ is the dynamic elastic modulus of the specimens after the nth freeze–thaw cycles (MPa), *E*_0_ is the initial dynamic modulus of elasticity of the specimens (MPa), *V*_n_ is the longitudinal wave velocity after the nth freeze–thaw cycles (km/s), *V*_0_ is the initial longitudinal wave velocity of the specimens (km/s), *L* is the initial length of the specimens (mm), *T*_n_ is the sound time of the specimens after the nth freeze–thaw cycles, and *T*_0_ is the initial sound of the specimens.

**Figure 18:**
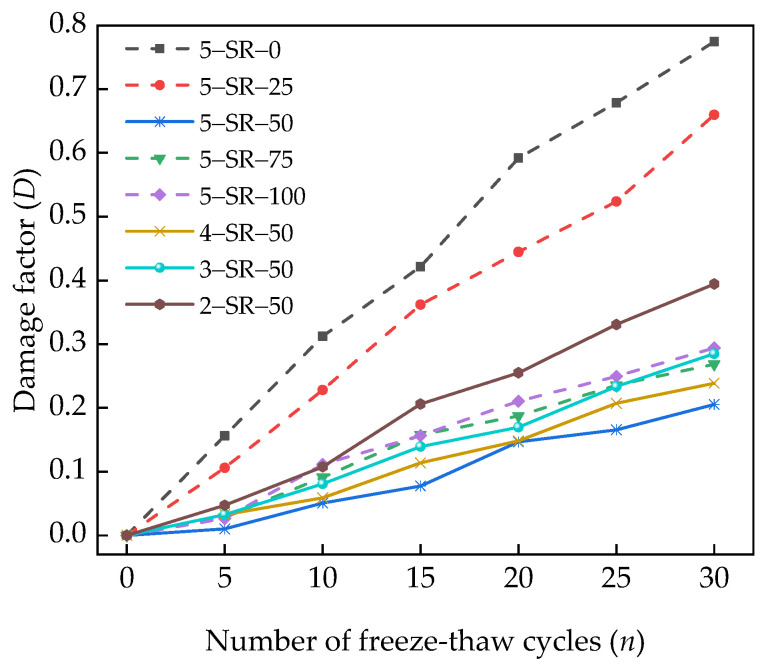
Relation between damage factor *D* and the number of freeze–thaw cycles *n*.

The relation between damage factor *D* and the number of freeze–thaw cycles *n* can be obtained using Equations (7) and (8), as shown in Figure 18.

Figure 18 shows that the damage factor, *D*, of the specimens increases with an increase in the number of freeze–thaw cycles. The damage caused to the specimens without slag is greater than that caused to samples with slag, and the freeze–thaw damage with low cement content is larger as compared to samples with high cement content. After 30 freeze–thaw cycles, the damage factors of 5-SR-25, 5-SR-50, 5-SR-75, and 5-SR-100 samples were 0.6599, 0.2056, 0.2686, and 0.2639, respectively. Compared with the 5-SR-0 sample, the freeze–thaw damage of 5-SR-25, 5-SR-50, 5-SR-75, and 5-SR-100 specimens decreased by 0.114, 0.569, 0.506, and 0.480, respectively. For a given cement content, with an increase in the slag replacement rate, the freeze–thaw damage of specimens decreased at first and then increased, and the freeze–thaw damage of 5-LZ-50 was the least. The proper amount of slag can form a relatively close accumulation system with cement, fly ash, and macadam, thereby increasing the compactness of the specimens. However, when the slag replacement rate was 50%, the appropriate and uniformly distributed closed pores resist a part of the water seepage pressure, effectively reducing the invasion speed of water, the damage of frost-heaving force, and osmotic pressure on the specimens. However, with the further increase in slag replacement rate, excessive closed pores will accelerate the connection and formation of capillaries, promote the invasion of water, increase the frost-heaving force caused by water condensation into ice, and reduce the frost resistance of the specimens. With an increase in cement content, the damage factor of the specimens decreases gradually. The main reason is that with an increase in cement content, the contents of C–S–H and other gel materials generated by the secondary hydration reaction in the specimens increase, thereby increasing the density of the interface transition zone, improving the stability and strength of the specimen, restraining the development and expansion of microcracks, and enhancing the frost resistance of the specimens.

### 4.3. Strength Degradation under Ultrasonic Testing

**Figure 19:**
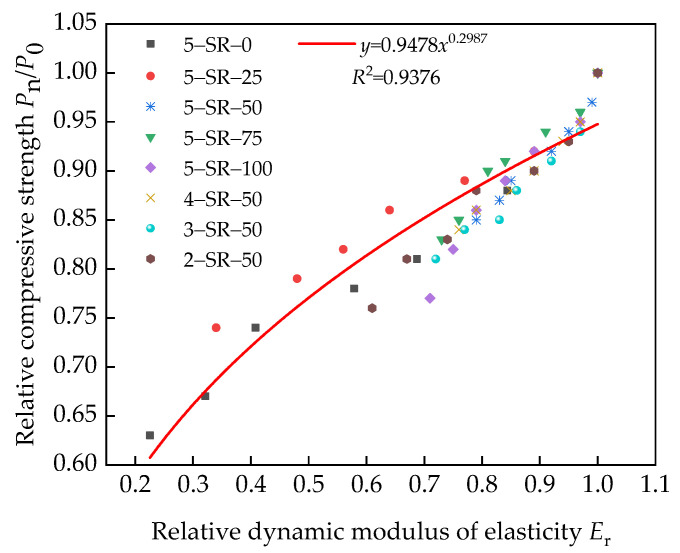
Relation between the relative dynamic elastic modulus and relative compressive strength of specimens.

Figure 19 shows that the growth rule of damage factor *D* is similar to the unconfined compressive strength, *D*_c_. Reference [37] shows that after the freeze–thaw cycles, the relation between the relative dynamic elastic modulus, *E*_r_, and the relative compressive strength is similar to the power function. Therefore, Equation (9) is introduced to evaluate the relationship between the relative dynamic elastic modulus and the relative compressive strength of the specimens.

(9) $$\frac{P_{n}}{P_{0}}=aE_{{}_{r}}^{b}$$

In the equation, the values of *a* and *b* are obtained by nonlinear regression analysis.

Figure 19 shows the fitting curve of relative dynamic elastic modulus and relative compressive strength.

According to the fitting results shown in Figure 19, Equation (10) can be obtained.

(10) $$\frac{P_{n}}{P_{0}}=0.9478E_{{}_{r}}^{0.2987}\;R^{2}=0.9376$$

Figure 19 shows that Equation (9) is in good agreement with the test results, indicating that a power function relationship exists between the relative dynamic elastic modulus and the relative compressive strength of the specimens. Therefore, ultrasonic testing can be used to detect the damage condition of unconfined compressive strength of the specimens under freeze–thaw cycles.

## 5. Discussion

Slag and macadam stabilized with cement and fly ash mixture is a new type of pavement base material. The main road performance indexes of the material were studied through mechanical, frost resistance, and ultrasonic tests. The results show that the mechanical and frost resistance properties of the mixture meet the requirements of Chinese pavement base specifications. Compared with previous studies, the paper mainly has the following two innovations:

(1) Macadam of 0–4.75 mm was replaced by 0–4.75 mm slag, which not only reduced the consumption of macadam and the pollution of slag caused to the environment but also significantly increased the frost resistance of the mixture.
(2) Based on the principles of ultrasonic testing, the relationship models between ultrasonic wave amplitude and unconfined compressive strength, freeze–thaw damage factor *D,* were established. According to the relationship models, ultrasonic nondestructive testing was used to detect the unconfined compressive strength and freeze–thaw damage degree of the mixture.

However, this study also has the following deficiencies, which are also the focus of future research:

(1) The fatigue and shrinkage performance of the mixture are not tested.
(2) The meso-finite element model of mixture is not established.
(3) A machine learning algorithm is not used to predict the strength and freeze–thaw damage degree of mixture.

Therefore, we will gradually improve these deficiencies in future studies.

## 6. Conclusions

According to the mechanical, frost resistance, and ultrasonic test results of slag and macadam stabilized with cement and fly ash mixture, the following conclusions are drawn:

(1) The optimum water content of the specimens increases, and the maximum dry density decreases with an increase in the slag replacement rate. Slag has an adverse effect on the unconfined compressive strength of the specimens. However, with extension of the curing age, the adverse effect of slag on the unconfined compressive strength of the specimens decreases gradually. With an increase in the cement content, the unconfined compressive strength of the specimens increases.
(2) The appearance of the specimens worsens, and the unconfined compressive strength decreases gradually with the increase of freeze–thaw cycles. The unconfined compressive strength loss of the specimens decreases at first and then increases with the increase of slag replacement rate, and the unconfined compressive strength loss decreases with the increase of cement content. When the cement content is 5% and the slag replacement rate is 50%, the unconfined compressive strength loss of the specimen is minimal.
(3) According to the test results, the relationship model between compressive strength and splitting strength of the mixture was established by linear function and power function regression analyses. The relationship model between unconfined compressive strength at 7 days and ultrasonic amplitude was established. The formulas between ultrasonic wave velocity *v*, relative dynamic elastic modulus *E*_r_, and damage factor *D* were proposed. The strength attenuation model between relative dynamic elastic modulus and relative compressive strength under freeze–thaw cycles was established to evaluate the frost resistance of the mixture.
(4) The comprehensive test results show that the slag replacement rate is recommended to be ~50%, and the mixture has good mechanical and frost resistance properties. When the cement content is 5%, the mixture can meet the technical requirements of expressway and first-class highway pavement base in China.

## Figures and Tables

**Table 1:** Chemical compositions of slag, fly ash, and cement.

Composition	SiO_2_	Al_2_O_3_	Fe_2_O_3_	CaO	MgO	K_2_O	Na_2_O	TiO_2_	SO_3_	LOI
Slag (%)	47.47	22.69	8.77	8.57	1.84	2.23	1.09	1.56	1.21	4.52
Fly ash (%)	45.16	32.91	7.47	4.95	1.20	2.12	0.78	1.77	0.80	2.63
Cement (%)	22.12	7.42	5.33	56.85	1.53	0.76	0.33	0.11	2.87	1.52

**Table 2:** Physical and mechanical properties of slag and macadam.

Material	Particle Size (mm)	Crushing Value (%)	Apparent Density (g·cm^−3^)	Packing Density (g·cm^−3^)	Water Absorption Rate (%)
Slag	0–2.36	/	2.353	0.786	11.1
	2.36–4.75	38.4	2.414	0.758	
Macadam	0–2.36	/	2.578	1.534	0.68
	2.36–4.75	16.7	2.622	1.507	
	4.75–9.5	18.8	2.679	1.432	0.64
	9.5–16	20.9	2.694	1.505	0.56
	16–26.5	21.5	2.705	1.536	0.51

**Table 3:** Main performance indices of cement.

Setting Time (min)		Flexural Strength (MPa)		Compressive Strength (MPa)		Fineness (%)
Initial Setting	Final Setting	3 Days	28 Days	3 Days	28 Days	
225	375	5.61	8.72	25.50	42.65	1.63

**Table 4:** Proportions of mixture components.

Mixture Number	Mass Fraction of Materials (%)						
	Gradation of Macadam (mm)				Slag (mm)	Fly Ash	Cement
	16–26.5	9.5–16	4.75–9.5	0–4.75	0–4.75		
5-SR-0	18	19	15	28	0	15	5
5-SR-25	18	19	15	21	7	15	5
5-SR-50	18	19	15	14	14	15	5
5-SR-75	18	19	15	7	21	15	5
5-SR-100	18	19	15	0	28	15	5
4-SR-*y*	18	19	16	28-*x*	*x*	15	4
3-SR-*y*	18	19	17	28-*x*	*x*	15	3
2-SR-*y*	18	19	18	28-*x*	*x*	15	2

Note: 5-SR-50 is used as an example to illustrate the numbering method of the mixture, where 5 indicates that the mass fraction of cement is 5%; SR-50 indicates a mass fraction 50% for the 0–4.75 mm slag replacing the 0–4.75 mm macadam; The value of *x* is determined by unconfined compressive strength tests as 5-SR-0 to 5-SR-100, *y* = 100*x*/28.

**Table 5:** Proportions of mixture parameters.

Mixture Number	Optimum Moisture Content (%)	Maximum Dry Density (g·cm^−3^)
5-SR-0	8.3	2.022
5-SR-25	9.3	1.970
5-SR-50	10.5	1.915
5-SR-75	11.8	1.858
5-SR-100	13.4	1.809

**Table 6:** Calculation results of strength representative value of 95% guarantee rate of after 7 days.

Mixture Number	Maximum Value (MPa)	Minimum Value (MPa)	Average Value (MPa)	Standard Deviation (%)	Variation Coefficient (%)	Representative Value of 95% (MPa)
5-SR-0	6.71	5.97	6.28	38.47	6.13	5.65
5-SR-25	6.13	5.63	5.93	26.57	4.48	5.49
5-SR-50	5.93	5.29	5.65	32.40	5.74	5.11
5-SR-75	5.78	5.05	5.37	39.03	7.28	4.72
5-SR-100	5.35	4.71	5.06	32.27	6.38	4.53
4-SR-50	5.75	4.71	5.21	51.98	9.97	4.36
3-SR-50	4.44	3.86	4.13	29.41	7.13	3.64
2-SR-50	3.01	2.63	2.79	20.12	7.21	2.46

**Table 7:** Ultrasonic test results of specimens before freeze–thaw cycles.

Mixture Number	Measured Distance	Sound Time	Wave Velocity
	*l* (mm)	*t* (μs)	*v* (m/s)
5-SR-0	151.6	39.8	3806.7
5-SR-25	151.8	41.5	3661.1
5-SR-50	151.3	41.9	3614.4
5-SR-75	150.9	44.0	3427.7
5-SR-100	151.5	45.1	3362.7
4-SR-50	151.2	43.1	3508.1
3-SR-50	151.3	46.9	3223.3
2-SR-50	151.7	52.6	2883.1

## Data Availability

The datasets generated and analyzed during the current study are available from the corresponding author upon reasonable request.
